# The Influence of Different Microstructure on Tensile Deformation and Acoustic Emission Behaviors of Low-Alloy Steel

**DOI:** 10.3390/ma13214981

**Published:** 2020-11-05

**Authors:** Wenbin Ma, Hongyun Luo, Zhiyuan Han, Linyan Zhang, Xiaoguang Yang

**Affiliations:** 1 School of Energy and Power Engineering, Beihang University, Beijing 100191, China; binwenma2008@126.com (W.M.); yxg@buaa.edu.cn (X.Y.); 2 School of Materials Science and Engineering, Beihang University, Beijing 100191, China; hzy19851227@163.com (Z.H.); linyanzhang2010@163.com (L.Z.); 3 Beijing Advanced Innovation Centre for Biomedical Engineering, Beihang University, Beijing 100191, China

**Keywords:** acoustic emission, tensile deformation, low-alloy steel, tempering treatment

## Abstract

The effect of different microstructures, obtained under different tempering temperatures on acoustic emission (AE) characteristics and source mechanisms during tensile deformation, was investigated in this study. Different heat treatments were carried out on hot-rolled low-alloy steels to obtain different microstructures (ferrite/pearlite, tempered martensite and tempered sorbite) and the AE was used to monitor the deformation and fracture process of samples of different types (BM, 200 °C tempered and 600 °C tempered). The results showed that the microstructure had different influences on the high amplitude burst-type signals and low amplitude continuous-type signals during the deformation and fracture process of low-alloy steels. In the 200 °C tempered sample, the continuous-type signals were enhanced by the high yield stress and dislocation velocity induced by the block of the lath martensite whose substructure was high-density dislocation. On the other hand, the interaction of the precipitates with the local dislocations increased the intensity of AE events, thus generating burst-type signals with higher amplitude in the 600 °C tempered samples.

## 1. Introduction

Low-alloy steels, such as C–Mn steels are extensively applied in building, bridges, vessels, pipelines and other fields due to their excellent mechanical properties, such as high strength, workability and weldability [1,2]. Due to the wide application of low-alloy steels, it is important to monitor the deformation and fracture behavior of the steels. Acoustic emission (AE) is a phenomenon in which transient elastic waves are generated by the rapid release of strain energy from localized sources in materials [3,4]. In past decades, AE had been widely employed as an effective non-destructive evaluation (NDE) technique [5] with high sensitivity [6,7] to detect the deformation and fracture process for many types of metals, such as low-alloy steels [2,3,8,9,10,11], aluminium alloy [12,13], and non-metals [14,15,16,17].

Low-alloy steels were often used in the different heat treatment states, which could obtain different microstructure and the characteristics of the AE signal were closely related to the microstructure of materials. It was of great interest in discussing the influence of microstructure on the AE signals during deformation and fracture process. Lots of previous studies had been conducted to investigate the AE response during the deformation and fracture process of steels with different microstructure. Wadley et al. [18,19,20] had reported the relationship of microstructure and acoustic emission in ferritic steels. During the tensile test, they found the AE signals of ferritic steels under different heat treatment burst near macro-yielding and inferred that the AE sources might be dislocation motion in the ferritic grains. However, they did not distinguish the types of AE signals in the whole deformation and fracture stages. Moorthy et al. [21] demonstrated that the microstructure had a strong influence on AE during stage 2 fatigue crack growth. They found that the high AE activity was attributed to the influence of cyclic plasticity, microcracking and crack closure phenomena in the weld specimen. Houssny-Emam et al. [22] reported that the AE activity of annealed specimens was higher than that of quenched-tempered specimens during a low-cycle fatigue test. Vinogradov [23] et al. reported that dislocation slip, twining and martensitic transformation were the AE sources during the deformation and fracture process of high-alloyed Cr–Mn–Ni cast steel with different nickel content and the clustering had been successfully used to identify the AE sources. In our previous work, some AE sources had been found in the deformation of Mg alloy and welding low-alloy steel. Han [24] et al. found that crack extension and twinning at the crack tip were two major AE sources during fatigue crack propagation in the Mg alloy. Chen [25] et al. demonstrated that the inclusions inside welds might provide an extra AE source. However, some important AE parameters, such as amplitude, energy, duration and waveforms, which could be used to confirm the AE source mechanisms associated with the dislocation dynamics in the Q345steel, were not clearly investigated in previous studies.

In this study, Q345 steel with different microstructure had been prepared through heat treatment. The tempering treatment could eliminate the residual stress and significantly affect the final mechanical properties for Q345 steel. Therefore, it was interesting to further study the mechanical properties of the tempered Q345 steel. The relationship among the tempering temperature, microstructure, and mechanical properties of Q345 steel was discussed. Tensile tests were carried out, and AE monitoring was performed simultaneously. The effect of different microstructure on AE and AE source mechanisms during deformation and the fracture process had been investigated.

## 2. Experimental Procedure

### 2.1. Materials and Heat-Treatment Process

As received hot-rolled Q345 steel (Chinese Code), which was 16 mm-thick plates in the standard heat treatment condition was used and marked as a base metal (BM) in this study. The chemical composition of Q345 steel was given in Table 1. In this work, the other two samples obtained by tempering treatment were also studied. Firstly, the other two samples were fully austenitized at 950 °C for 1 h, then water quenched to room temperature. After that, tempering treatment was conducted at 200 °C and 600 °C for 1 h, respectively, to eliminate residual stress and improve toughness [26] and water cooled to room temperature. The two samples were named the 200 °C tempered sample and 600 °C tempered sample, respectively. The surface of the three samples (BM, 200 °C tempered and 600 °C tempered) was mechanically grounded up to 2000 grit and polished with SiC abrasive paper. The polished surface was subsequently etched with a 3 vol% alcohol nitrate solution. The microstructure of the three samples were observed by an optical microscope (OM, Leica DM4000, Leica Microsystems, Weztlar, Germany). The fracture morphologies were observed using an electron scanning electron microscope (SEM, JSM-5800, Japan Electronics, Tokyo, Japan).

### 2.2. Tensile Test and Acoustic Emission Monitoring

**Figure 1:**
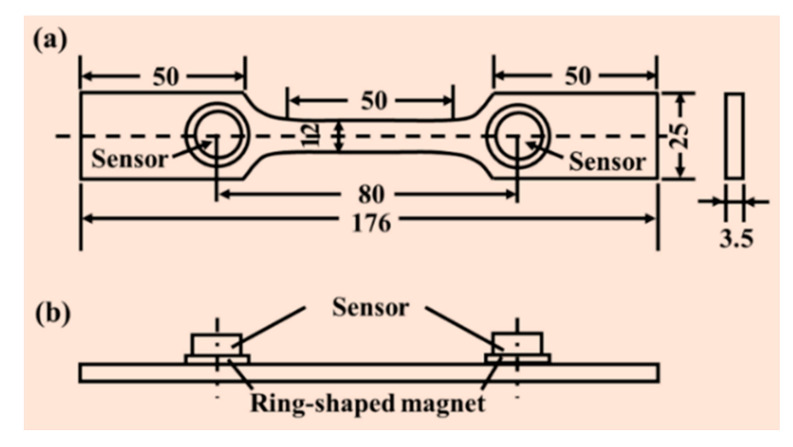
(**a**) Details of tensile samples; (**b**) sensors’ arrangement.

The tensile tests of three types of samples were carried out on an electronic universal testing machine (SANSI, Shenzhen Sansi Testing Technology Corporation, Shenzhen, China) at room temperature. The tensile axis was parallel to the rolling direction [27]. Nominal strain rate was 6.68 × 10^−4^ s^−1^. The shape and dimensions of the tensile samples were designed according to the GB/T228-2002 national standard (Figure 1a). At least three parallel samples for each type were tested to confirm the accuracy of the test.

During tensile tests, a digital signal processor with an AEwin v2.19 AE system (Physical Acoustic Corporation, Princeton, NJ, USA) was used to record and analyze AE signals. The AE signals were captured by the broadband piezoelectric transducers with a resonant frequency of 375 kHz, a preamplifier with 40 dB gain and a compatible filter (10 kHz–2 MHz). The positions of AE sensors could be found in Figure 1b. The sensors were attached to the sample with a ring-shaped magnet. Vaseline was used to improve the signal transmission efficiency at the samples/sensors interface. The AE energy and average frequency thresholds were optimized to eliminate noises from the external environment [27].

## 3. Results and Discussion

### 3.1. Microstructure Characterization

**Figure 2:**
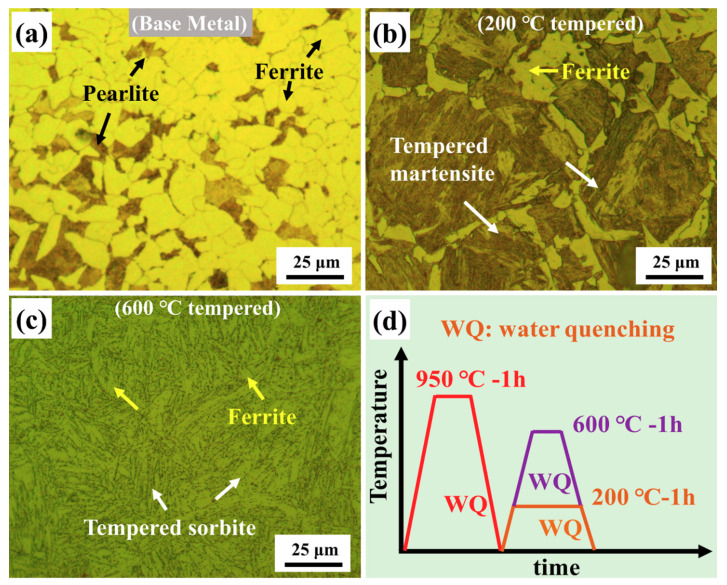
Optical microscopy observations of the microstructure of different samples: (**a**) base metal sample; (**b**) 200 °C tempered sample; (**c**) 600 °C tempered sample and (**d**) Schematic illustration of heat treatment.

The samples with different microstructure are displayed in Figure 2. It can be seen in Figure 2a that the BM sample consisted of typical ferrite and pearlite. The average grain size of the ferrite was about 15 µm. The fraction of ferrite phase was 58%, which was calculated using the image J software. It was noticeable from Figure 2b that the typical microstructure of the 200 °C tempered sample was tempered martensite, which consisted of lath martensite with high dislocation density as its substructure [28,29,30] and a small amount of retained ferrites. The fraction of tempered martensite phase was 74%. As shown in Figure 2c, after 600 °C tempering, the microstructure of the sample was composed of the ferritic matrix and uniformly dispersed fine granular carbide precipitates, which is known as tempered sorbite [31,32]. The fraction of tempered sorbite phase was 82%.

### 3.2. Tensile Performance and Fracture Morphology

**Figure 3:**
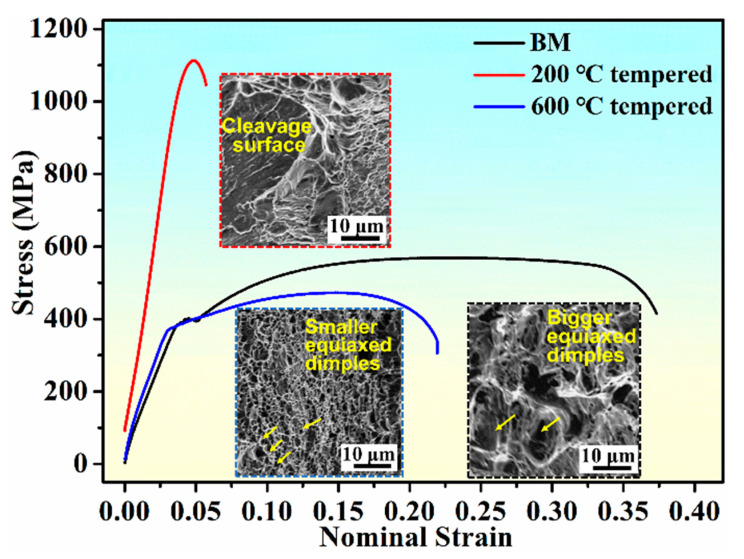
Stress-nominal strain curves. BM curve was adapted from Han [27].

The tensile test results of BM, 200 °C tempered and 600 °C tempered samples at room temperature are shown in Figure 3 and Table 2. Zhang et al. [33] reported that the change of elongation with tempering temperature is contrary to that of the strength. The same result was also found in this study, as shown in Figure 3. The stress-nominal strain curves demonstrated that different heat-treated samples had different tensile performances. The yield strength was determined by the method of the 0.2% offset plastic strain [34]. It was found that the 200 °C tempered sample had the highest strength with yield strength of 850 MPa and ultimate strength of 1138 MPa, which resulted from high density dislocation and dislocation motion restricted by lath martensite boundaries [35,36,37,38]. As shown in Table 2, the yield strength of 600 °C tempered sample was 380 MPa, which was a little higher than that of the BM sample. This was attributed to the effects of precipitation strengthening due to the carbide precipitates distributed in the ferritic matrix. The BM sample had the largest strain (∼37%), clearly higher than that of the 200 °C tempered sample (less than 6%) and 600 °C tempered sample (less than 25%), and showed the best plasticity. For the 200 °C tempered sample, the dislocations started and slipped with difficulty, thus the elongation was the lowest with almost no work-hardening. Moreover, obvious yielding platform could be found in BM sample, which showed typical discontinues yielding processes. Nevertheless, obvious yielding platforms could hardly be found during the fracture process of 200 °C tempered and 600 °C tempered samples, indicating clearly continuous yielding processes (Figure 3). It was found that as the tempering temperature increased, the elongation increased and the strength decreased. According to the above discussion, it was known that the decrease in strength could be prevented by tempering at lower temperature (200 °C) [39].

**Figure 4:**
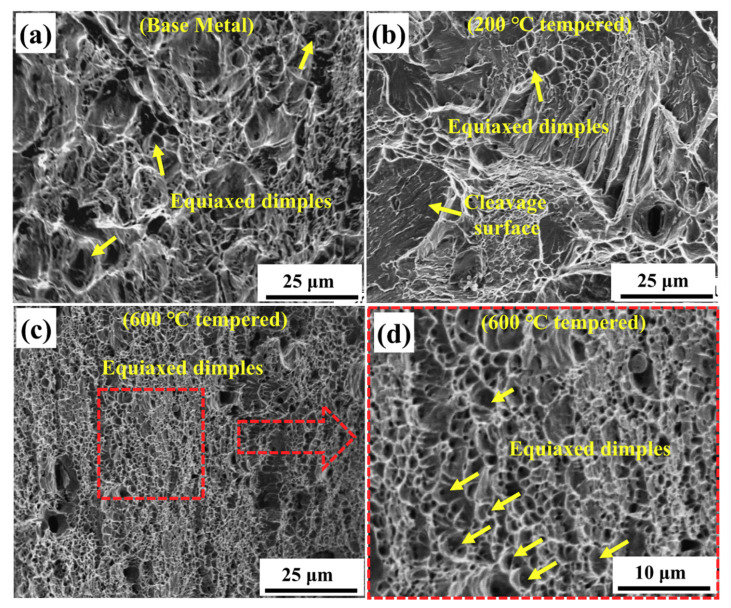
Morphologies of the fracture surfaces (**a**) BM sample; (**b**) 200 °C tempered sample and (**c**,**d**) 600 °C tempered sample with different magnifications.

As shown in Figure 4, the morphologies on the fracture surfaces of the three types of sample were significantly different. It could be seen from Figure 4a that equiaxed dimples with different sizes and depths could be found on the fracture surface of the BM sample and the average dimple size was 2.5 μm. Large number of dimples were observed, indicating that the BM sample was the ductile fracture due to the growth and coalescence of voids [34,40]. Both cleavage planes and ductile dimples were observed in the fracture surface of the 200 °C tempered sample. The fracture morphology of the 200 °C tempered sample showed the mixed characteristics of brittle cleavage and dimple fracture behavior (Figure 4b). The facet sizes of the cleavage were consistent with the tempered martensite areas observed in Figure 2b. The relatively flat fracture surface implied that the 200 °C tempered sample had poor plasticity, which was consistent with the corresponding stress–nominal strain curve displayed in Figure 3. Clearly, as shown in Figure 4d, compared with BM sample, lots of smaller and shallower dimples could be seen on the fracture surface of 600 °C tempered sample and the average dimple size was less than 1 μm, which indicated the lower elongation than the BM sample [41]. The growing process of dimples might cost more energy [25], so the 600 °C tempered sample had better tensile plasticity performance than the 200 °C tempered sample.

### 3.3. Acoustic Emission Results during Deformation and Fracture Process

#### 3.3.1. Acoustic Emission (AE) Waveforms and Amplitude during Deformation and Fracture Process

**Figure 5:**
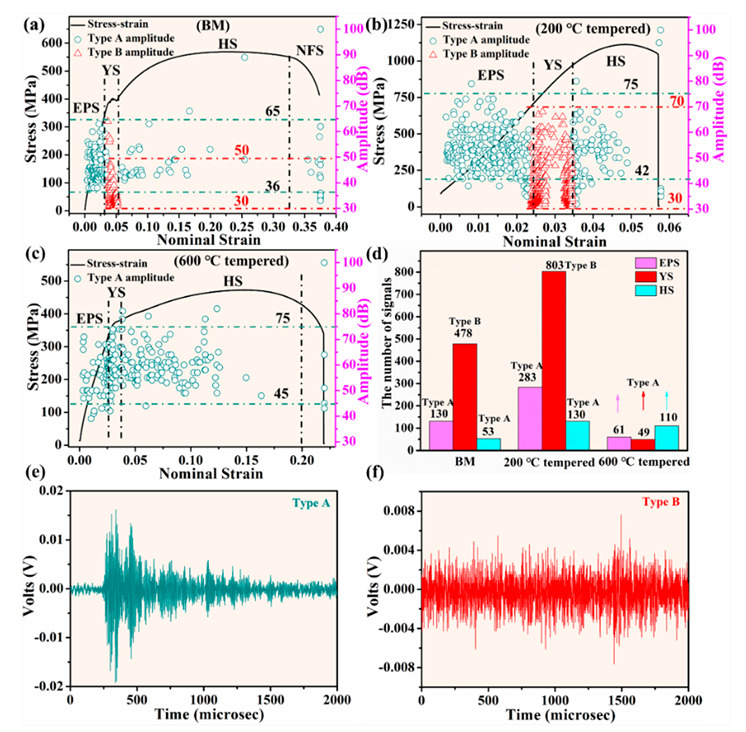
The acoustic emission (AE) amplitude and stress versus nominal strain of different samples, (**a**) BM sample [27]; (**b**) 200 °C tempered sample; (**c**) 600 °C tempered sample; (**d**) Number of signals; AE signal waveforms, (**e**) burst-type signal (type A) and (**f**) continuous-type signal (type B), adopted from [27].

As shown in Figure 5, during the deformation and fracture process of BM, 200 °C tempered and 600 °C tempered samples, two types of AE signals which were named as type A signal with a burst-type waveform (Figure 5e) and type B signal with a continuous-type waveform (Figure 5f) were mainly observed. Type A signals were produced by dislocation multiplication and unpinning from Cottrell atmospheres or dislocation tangling. On the other hand, type B signals were attributed to the collective synergistic motion of high-density dislocations [27]. As shown in Figure 5, the strain–stress curves of the three types of samples during the deformation and fracture process can be divided into four stages. The first is the stage of elastoplastic deformation process (EPS). The second is the yield stage (YS) and the third is the strain hardening stage (HS). The fourth stage is the stage of the necking and fracture process (NFS) [10,27]. The YS of the BM and 200 °C tempered samples was determined according to the range of type B signals which appeared around the yield point (Figure 5). The YS of the 600 °C tempered sample was determined according to the range of the steps where the cumulative energy sharply increased around the yield point.

Figure 5a,b shows that the amplitude of the type A signal was high, which was mainly distributed in the EPS and HS for BM and 200 °C tempered samples. The type B signals were only distributed in the YS of BM and the 200 °C tempered samples, respectively. For the 600 °C tempered sample, only type A signals could be observed in the whole stages during the deformation and fracture process and there were no type B signals detected (Figure 5c). According to type A signals, the amplitude distribution of different microstructure was different. The amplitude of type A signals in the BM sample was generally between 36–65dB during the whole deformation and fracture process, and it was mainly below 55 dB. In the 200 °C tempered sample, the amplitude of type A signals was mainly between 42–75 dB. However, the amplitude of the 600 °C tempered sample was mostly distributed between 45–75 dB. The AE parameter and stages of occurrence of waveforms for different samples are summarized in Table 3. The number of type A and type B signals at different fracture stages of BM, 200 °C tempered and 600 °C tempered samples, are shown in Figure 5d. The 200 °C tempered sample had the larger amount of type B signals in the yield stage, which was two times that of the BM sample due to the high-density dislocation existing in the lath martensite.

#### 3.3.2. Acoustic Emission (AE) Energy during Deformation and Fracture Process

**Figure 6:**
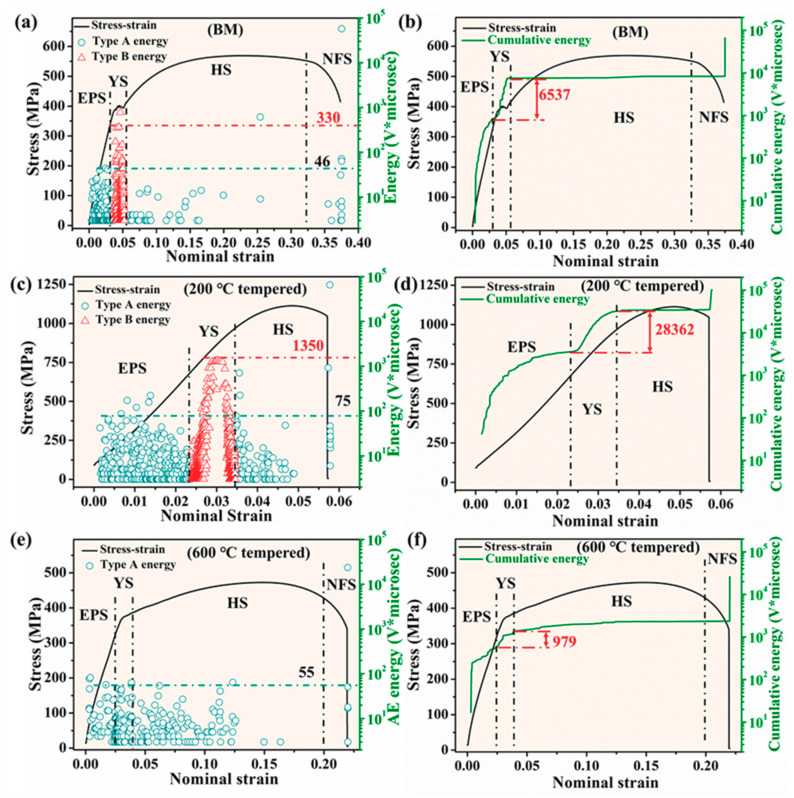
AE energy and cumulative AE energy versus nominal strain of different types of samples, (**a**,**b**) BM sample; (**c**,**d**) 200 °C tempered sample; (**e**,**f**) 600 °C tempered sample.

The relationships of AE energy and stress with nominal strain of different samples are displayed in Figure 6a,c,e. For all samples tested, the AE energy increased rapidly at the onset of plastic yielding and reduced gradually afterwards. Vinogradov et al. [42,43] observed a similar phenomenon about the change of AE power during a tensile test.

The AE energy values of different samples were quite different. In the elastoplastic stages, most AE energy values for BM and 200 °C tempered samples were less than 46 V∙μs and 75 V ∙μs, respectively. It can be seen from Figure 6 that significant AE was generated in the region near the macroscopic yielding for all the specimens. This phenomenon was also reported by Barat et al. [44]. There was no obvious yield point on the stress-strain curve for the 200 °C tempered sample, which was consistent with many initial dislocations existing before deformation [18]. In the yield stages, most AE energy values for BM and 200 °C tempered samples were less than 330 V μs and 1350 V∙μs, respectively. In the whole deformation and fracture process, most AE energy values for 600 °C tempered samples were less than 55 V∙μs. The AE energy values of all samples gradually decreased during the hardening stage and then increased sharply in the final fracture.

The cumulative AE energy was also used to show fracture mechanisms and behaviors of BM, 200 °C tempered and 600 °C tempered samples, respectively. As shown in Figure 6b,d,f, the cumulative energy of all samples increased sharply in the elastoplastic stage and yield stage, respectively, and showed two steps. In the yield stage, the cumulative AE energy of 600 °C tempered sample was obviously lower than that of the BM and 200 °C tempered samples because of the lack of type B signals. The cumulative energy of the 200 °C tempered sample was 2.83 × 104 V∙μs, which was more than one order of magnitude higher than that of the 600 °C tempered sample (979 V∙μs) and four times as much as the BM sample (6.54 × 103 V∙μs).

The above results indicated that the deformation mechanisms were quite different among BM, 200 °C tempered and 600 °C tempered samples, which might be related to the microstructure of samples. The 200 °C tempered sample had far more AE energy released than BM sample during deformation and fracture processes and more type B signals were detected in the yield stage.

#### 3.3.3. The Duration and Acoustic Emission (AE) Counts During Deformation and Fracture Process

**Figure 7:**
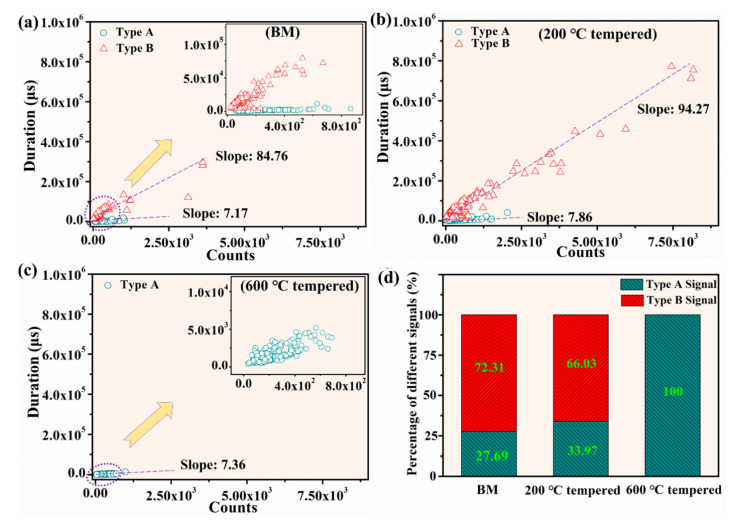
Duration versus Counts for (**a**) BM sample; (**b**) 200 °C tempered sample; (**c**) 600 °C tempered sample and (**d**) percentage of different type of signals.

In order to further demonstrate the effect of different microstructure (ferrite/pearlite, tempered martensite and tempered sorbite) on AE behaviors during the deformation and fracture process, a multi-parametric analysis of AE data was undertaken, and the results are shown in Figure 7. Figure 7 illustrates the plot of counts versus duration of individual AE event for different samples. Duration was one of the acoustic parameters, which stood for the time difference between the first and last threshold crossings [5]. It could be seen from Figure 7a that duration was distributed between 0 and 3 × 10^5^ μs, and the counts ranged from 0 to 3.6 × 10^3^ for BM sample. For 200 °C tempered sample, the duration and counts were mainly in ranges of 0 to 8 × 10^5^ μs and 0 to 8.2 × 10^3^, respectively (Figure 7b). By contrast, for the 600 °C tempered sample, the duration and counts were mainly in the ranges of 0 to 5.2 × 10^3^ μs and 0 to 7.0 × 10^2^, respectively (Figure 7c). The duration and counts usually reflect the nature of AE source mechanisms. According to the analysis above, the 200 °C tempered sample had higher distributions of duration and counts than BM and 600 °C tempered samples. It was reasonably assumed that the main AE source for 200 °C tempered martensite specimen was the cleavage fracture events rather than plastic events during deformation and fracture processes. It is shown in Figure 7a,b that type A and type B signals obviously distributed in the different region and had different slope values. According to the discussion above, it was known that the type (ductile or brittle) of the deformation and fracture could be distinguished by using the nature of variation between duration with counts. Moreover, the percentage of type A and type B signals during the whole deformation and fracture processes in BM, 200 °C tempered and 600 °C tempered samples were counted, respectively. As shown in Figure 7d, more than 66% of all signals generated in BM and 200 °C tempered samples were type B signals, while type A signals only accounted for 27.69% of all signals in the BM sample.

#### 3.3.4. Effects of Microstructure on Burst-Type Signals

As shown in Figure 5, different microstructures had different influences on the type A signals. The type A signals generated during the deformation and fracture process of the 200 °C tempered sample had higher amplitude (~75 dB) than the BM sample (~65 dB). This might be related to the local dislocation avalanche caused by the high-density dislocation in lath martensite. The dislocation velocity in the local dislocation avalanche was also higher than the local dislocation event in the BM sample, so it had a higher amplitude distribution. Moreover, the amplitude distribution in the 600 °C tempered sample was higher than that of the BM sample and there were more high-amplitude signals, which indicated that the local dislocation events were strengthened. A reasonable explanation was that the interaction between the precipitates in the sample and the local dislocation events enhanced the stress and dynamics of the relevant dislocation events. Therefore, the intensity, or amplitude, of the local dislocation event might be improved, and a higher amplitude distribution appeared in the 600 °C tempered sample. Mukhopadhyay [45] et al. also demonstrated that the precipitate particles in the M250 grade martensite steel played an important role in strengthening material and then improved amplitude distribution. Compared with the other two samples, the smaller dimples (less than 1 μm) could be seen in the fracture surface of the 600 °C tempered sample, which indicated that stress concentration existed in the material around the precipitates, and dimples firstly nucleated at these locations. At the same time, these fracture characteristics also indicated that in the hardening stage there might be early tearing of the interdimple ligament and separation of the precipitates from the matrix, which might become a relatively strong AE source and produce a type A signal with high amplitude.

#### 3.3.5. Effects of Microstructure on Continuous-Type Signals

The effects of microstructure on low-amplitude continuous-type signals were also very interesting. The initial dislocation density was much lower in the ferrite/pearlite microstructure [18]. During the deformation and fracture process of the BM sample, the generation of type B signals were directly related to dislocation activities during Luders band propagation [27]. However, as could be seen in Figure 6b, there was far higher type B energy observed in the 200 °C tempered sample which had continuous yield behavior. This indicated that Luders band propagation was not a necessary condition for the appearance of type B signal. In the 200 °C tempered sample, the amplitude of type B signal was higher than that of BM sample. It can be explained by the fact that due to the high density of initial dislocation in lath martensite, the slip of dislocation was very difficult, which led to a large number of dislocation pile-ups and produced high yield stress. Once the stress reached critical value, the dislocations overcame resistance and began to slip, which enhanced the dislocation avalanche effect. Many plugged dislocations had good motion consistency. Therefore, the AE signals generated in the yield stage had consistent phase, making the amplitude superposition easy [19]. The strength of the type B signal was enhanced due to the high dislocation velocity, caused by the high yield stress. As for the 600 °C tempered sample, dislocation motion might be impeded by dispersed precipitates, which decreased dislocation free path and velocity. Thus, type B signals could not be found in the 600 °C tempered sample.

## 4. Conclusions

This paper investigated the influence of different microstructures (ferrite/pearlite, tempered martensite and tempered sorbite) obtained by different heat treatment on plastic deformation and AE signals of Q345 steel. Combining the analysis of the fracture surfaces, tensile properties of Q345 steel with different microstructure, AE waveform (burst-type and continuous-type) and AE parameters (amplitude, energy, duration and counts) obtained by real-time AE monitoring, the following conclusions can be drawn:

Mainly two types of AE signal (type A and type B signals) were obtained during the deformation and fracture process of BM sample which consists with typical ferrite and pearlite. According to the statistics of the number of type A and type B signals, it was found that type B signals account for about 72.31%. It could be assumed that the AE source related to type B signals play a dominant role in the deformation and fracture process.

The typical microstructure of the 200 °C tempered sample is tempered martensite, which consists of lath martensite with high dislocation density as its substructure. There are also mainly type A and type B signals in the deformation and fracture process. The type B signals, which account for about 66.03% of the total signals are detected in the yield stage. It can be inferred that collective synergistic motion of dislocations related to type B signals is the main AE source mechanism.

The microstructure of the 600 °C tempered sample is composed of ferritic matrix and uniformly dispersed fine granular carbide precipitates, which is known as tempered sorbite. For the 600 °C tempered sample, only type A signals could be detected in the whole stages during the deformation and fracture process. It might be inferred that dislocation motion is the main AE source mechanism during the deformation and fracture process.

Type A signals are mainly distributed in the elastoplastic and hardening stages for BM and 200 °C tempered samples. The type B signals are only distributed in the yield stage of BM and the 200 °C tempered samples, respectively. In the yield stage, the cumulative AE energy in the 200 °C tempered sample is four times that of the BM sample, which might result from high density dislocation existing in the lath martensite. Moreover, in the yield stage, the AE energy of the 600 °C tempered sample is obviously lower than that of the BM and 200 °C tempered samples which might be due to the lack of type B signals. During the deformation and fracture process, the BM and 600 °C tempered samples exhibit ductile fracture characteristics and the 200 °C tempered sample mainly exhibits brittle cleavage fracture characteristics due to the existence of tempered martensite.

## Figures and Tables

**Table 1:** Chemical compositions (wt.%) of Q345 steel.

C	Mn	Si	P	S	Ca	Fe
0.16	1.42	0.31	0.022	0.033	0.10	balance

**Table 2:** Tensile properties of Q345 steel with different heat treatments.

Samples	Yield Stress /MPa	Ultimate Tensile Stress /MPa	Elongation
Base Metal	368	569	0.373
200 °C tempered	850	1138	0.0571
600 °C tempered	380	472	0.219

**Table 3:** AE parameter and waveforms for different samples. ^a^

Samples	Waveform Types	Amplitude (dB)	Appearing Stages
**BM**	Type A	36–65	EPS and HS
	Type B	30–50	YS
**200 °C tempered**	Type A	42–75	EPS and HS
	Type B	30–70	YS
**600 °C tempered**	Type A	45–75	EPS, YS and HS

^a^ EPS—the elastoplastic deformation stage; HS—the strain hardening stage; YS—the yield stage.

## References

[1] Wang H.W., Li W.T., Bai H., Xiao H.Q., Yu H.M. (2013). Failure Analysis of Q345 Steel Structures on Port Cranes. Appl. Mech. Mater..

[2] Xu J., Sun T., Xu Y., Han Q. (2020). Fracture toughness research of G20Mn5QT cast steel based on the acoustic emission technique. Constr. Build. Mater..

[3] Mukhopadhyay C., Sasikala G., Jayakumar T., Raj B. (2012). Acoustic emission during fracture toughness tests of SA333 Gr.6 steel. Eng. Fract. Mech..

[4] Wadley H., Mehrabian R. (1984). Acoustic emission for materials processing: A review. Mater. Sci. Eng..

[5] Jiang Z., Sherif M.M., Xing G., E Ozbulut O. (2020). Tensile characterization of graphene nanoplatelets (GNP) mortar using acoustic emissions. Mater. Today Commun..

[6] Chen J., Kan Q., Li Q., Yin H. (2019). Effects of grain size on acoustic emission of nanocrystalline superelastic NiTi shape memory alloys during fatigue crack growth. Mater. Lett..

[7] Illkova K., Dobroň P., Chmelik F., Kainer K.U., Balík J., Yi S., Letzig D., Bohlen J. (2014). Effect of aluminium and calcium on the microstructure, texture, plastic deformation and related acoustic emission of extruded magnesium–manganese alloys. J. Alloys Compd..

[8] Mukherjee P., Barat P., Jayakumar T., Kalyanasundaram P., Rajagopalan C., Raj B. (1997). Acoustic emission studies on welded and thermally treated AISI 304 stainless steel during tensile deformation. Scr. Mater..

[9] Roy H., Parida N., Sivaprasad S., Tarafder S., Ray K. (2008). Acoustic emissions during fracture toughness tests of steels exhibiting varying ductility. Mater. Sci. Eng. A.

[10] Akbari M., Ahmadi M. (2010). The application of acoustic emission technique to plastic deformation of low carbon steel. Phys. Procedia.

[11] Lyasota I., Kozub B., Gawlik J. (2019). Identification of the tensile damage of degraded carbon steel and ferritic alloy-steel by acoustic emission with in situ microscopic investigations. Arch. Civ. Mech. Eng..

[12] Scruby C., Wadley H., Sinclair J.E. (1981). The origin of acoustic emission during deformation of aluminium and an aluminium–magnesium alloy. Philos. Mag. A.

[13] Sun C., Zhang W., Ai Y., Que H. (2015). Study of the Tensile Damage of High-Strength Aluminum Alloy by Acoustic Emission. Metals.

[14] Suresh S., Moorthi N.S.V., Vettivel S.C., Selvakumar N., Jinu G.R. (2014). Effect of graphite addition on mechanical behavior of Al6061/TiB2 hybrid composite using acoustic emission. Mater. Sci. Eng. A.

[15] Anand Partheeban C.M., Rajendran M., Vettivel S.C., Suresh S., Moorthi N.S.V. (2015). Mechanical behavior and failure analysis using online acoustic emission on nano-graphite reinforced Al6061–10TiB_2_ hybrid composite using powder metallurgy. Mater. Sci. Eng. A.

[16] Chen G., Luo H., Yang H., Zhang T., Li S. (2018). Water effects on the deformation and fracture behaviors of the multi-scaled cellular fibrous bamboo. Acta Biomater..

[17] Rangel-Hernández V., Fang Q., Babelot C., Lohoff R., Blum L. (2020). An experimental investigation of fracture processes in glass-ceramic sealant by means of acoustic emission. Int. J. Hydrogen Energy.

[18] Wadley H.N.G., Scruby C.B. (1991). Cooling rate effects on acoustic emission-microstructure relationships in ferritic steels. J. Mater. Sci..

[19] Scruby C.B., Wadley H.N.G. (1993). Tempering effects on acoustic emission-microstructural relationships in ferritic steels. J. Mater. Sci..

[20] Wadley H.N.G., Scruby C.B. (1993). Spheroidal inclusion effects on acoustic emission-microstructural relations in ferritic steels. J. Mater. Sci..

[21] Moorthy V., Jayakumar T., Raj B. (1996). Influence of microstructure on acoustic emission behavior during stage 2 fatigue crack growth in solution annealed, thermally aged and weld specimens of AISI type 316 stainless steel. Mater. Sci. Eng. A.

[22] Houssny-Emam M., Bassim M. (1983). Study of the effect of heat treatment on low cycle fatigue in AISI 4340 steel by acoustic emission. Mater. Sci. Eng..

[23] Vinogradov A., Lazarev A., Linderov M.L., Weidner A., Biermann H. (2013). Kinetics of deformation processes in high-alloyed cast transformation-induced plasticity/twinning-induced plasticity steels determined by acoustic emission and scanning electron microscopy: Influence of austenite stability on deformation mechanisms. Acta Mater..

[24] Han Z., Luo H., Sun C., Li J., Papaelias M., Davis C. (2014). Acoustic emission study of fatigue crack propagation in extruded AZ31 magnesium alloy. Mater. Sci. Eng. A.

[25] Chen G., Luo H., Yang H., Han Z., Lin Z., Zhang Z., Su Y. (2019). Effects of the welding inclusion and notch on the fracture behaviors of low-alloy steel. J. Mater. Res. Technol..

[26] Li S., Zhu G., Kang Y. (2016). Effect of substructure on mechanical properties and fracture behavior of lath martensite in 0.1C–1.1Si–1.7Mn steel. J. Alloys Compd..

[27] Han Z., Luo H., Wang H. (2011). Effects of strain rate and notch on acoustic emission during the tensile deformation of a discontinuous yielding material. Mater. Sci. Eng. A.

[28] Su G., Gao X., Yan T., Zhang D., Cui C., Du L., Liu Z., Tang Y., Hu J. (2018). Intercritical tempering enables nanoscale austenite/ε-martensite formation in low-C medium-Mn steel: A pathway to control mechanical properties. Mater. Sci. Eng. A.

[29] Man T., Liu T., Ping D., Ohmura T. (2018). TEM investigations on lath martensite substructure in quenched Fe-0.2C alloys. Mater. Charact..

[30] Stormvinter A., Hedström P., Borgenstam A. (2011). Investigation of Lath and Plate Martensite in a Carbon Steel. Solid State Phenom..

[31] Jing G., Huang W., Yang H., Wang Z. (2020). Microstructural evolution and mechanical properties of 300M steel produced by low and high power selective laser melting. J. Mater. Sci. Technol..

[32] Liu F., Lin X., Song M., Yang H., Song K., Guo P., Huang W. (2016). Effect of tempering temperature on microstructure and mechanical properties of laser solid formed 300M steel. J. Alloys Compd..

[33] Zhang Y., Zhan D., Qi X., Jiang Z. (2019). Effect of tempering temperature on the microstructure and properties of ultrahigh-strength stainless steel. J. Mater. Sci. Technol..

[34] Jiao Z., Luan J., Zhang Z., Miller M., Ma W., Liu C. (2013). Synergistic effects of Cu and Ni on nanoscale precipitation and mechanical properties of high-strength steels. Acta Mater..

[35] Shamsujjoha M. (2020). Evolution of microstructures, dislocation density and arrangement during deformation of low carbon lath martensitic steels. Mater. Sci. Eng. A.

[36] Galindo-Nava E., Rivera-Díaz-Del-Castillo P. (2015). A model for the microstructure behaviour and strength evolution in lath martensite. Acta Mater..

[37] Du C.C., Hoefnagels J.J., Vaes R.R., Geers M.M. (2016). Block and sub-block boundary strengthening in lath martensite. Scr. Mater..

[38] Shibata A., Nagoshi T., Sone M., Morito S., Higo Y. (2010). Evaluation of the block boundary and sub-block boundary strengths of ferrous lath martensite using a micro-bending test. Mater. Sci. Eng. A.

[39] Zurnadzhy V., Efremenko V., Wu K., Azarkhov A., Chabak Y., Greshta V., Isayev O., Pomazkov M. (2019). Effects of stress relief tempering on microstructure and tensile/impact behavior of quenched and partitioned commercial spring steel. Mater. Sci. Eng. A.

[40] Luo H., Wang X., Liu Z., Yang Z. (2020). Influence of refined hierarchical martensitic microstructures on yield strength and impact toughness of ultra-high strength stainless steel. J. Mater. Sci. Technol..

[41] Zare A., Ekrami A. (2011). Influence of martensite volume fraction on tensile properties of triple phase ferrite–bainite–martensite steels. Mater. Sci. Eng. A.

[42] Vinogradov A., Yasnikov I., Merson D. (2019). Phenomenological approach towards modelling the acoustic emission due to plastic deformation in metals. Scr. Mater..

[43] Vinogradov A., Merson D., Patlan V., Hashimoto S. (2003). Effect of solid solution hardening and stacking fault energy on plastic flow and acoustic emission in Cu–Ge alloys. Mater. Sci. Eng. A.

[44] Barat K., Bar H., Mandal D., Roy H., Sivaprasad S., Tarafder S. (2014). Low temperature tensile deformation and acoustic emission signal characteristics of AISI 304LN stainless steel. Mater. Sci. Eng. A.

[45] Mukhopadhyay C., Rajkumar K.V., Jayakumar T., Raj B. (2009). Study of tensile deformation behaviour of M250 grade maraging steel using acoustic emission. J. Mater. Sci..

